# Can Platelet Indices Reduce Negative Appendectomy Rates?

**DOI:** 10.7759/cureus.4293

**Published:** 2019-03-21

**Authors:** Yavuz Yigit, Serkan Yilmaz, Asim E Ozbek, Onur Karakayali, Bilen Cetin, Huseyin C Halhalli

**Affiliations:** 1 Emergency Medicine, University of Health Sciences, Kocaeli Derince Training and Research Hospital, Kocaeli, TUR; 2 Emergency Medicine, Kocaeli University, Kocaeli, TUR; 3 Emergency Medicine, Kilis State Hospital, Kilis, TUR

**Keywords:** appendicitis, mean platelet volume, blood cell count

## Abstract

Introduction

The role of whole blood count parameters in the diagnosis of diseases in which inflammatory processes play a role is one of the more frequently mentioned topics in the literature in recent years. Studies of acute appendicitis have also been carried out in this regard, but studies focused on platelet parameters are few and contradictory. We aimed to investigate the role of mean platelet volume (MPV) and platelet distribution width (PDW) in the diagnosis of acute appendicitis.

Materials and methods

We retrospectively screened the medical records of patients older than 15 years who had an appendectomy from January 2012 to January 2015 at a training hospital in Kocaeli, Turkey. Patients were divided into three groups according to their pathology results: non-appendicitis (Group 1), uncomplicated appendicitis (Group 2), and complicated appendicitis (Group 3). We calculated the sensitivity, specificity, positive and negative predictive values, the likelihood ratios in the diagnosis of appendicitis for white blood cell (WBC), neutrophil count, c-reactive protein (CRP), MPV, and PDW values were calculated.

Results

There were no significant differences in the MPV between Group 1 (n = 39; 7.89 ± 1.32 fL), Group 2 (n = 119; 7.80 ± 1.19 fL), and Group 3 (n = 89; 7.70 ± 0.80 fL; p = 0.141). Also, we found no significant differences in PDW between Group 1 (117.38% ± 1.17), Group 2 (17.17% ± 1.04), and Group 3 (17.12% ± 0.64; p = 0.228).

Conclusions

Only nine of the 208 patients whose pathology reports confirmed appendicitis had healthy values for both CRP and WBC. Many factors affect MPV and PDW. Therefore, platelet indices are not useful markers in diagnosing acute appendicitis.

## Introduction

Acute appendicitis is the most common cause of urgent abdominal surgery, and the overall lifetime risk of developing appendicitis is 7% [1]. The diagnosis is usually established on the clinical floor, but symptoms and findings may not always be typical; this makes diagnosis difficult. Early diagnosis and treatment are important because delayed diagnosis can lead to perforation, which may increase the incidence of mortality and morbidity [2]. On the other hand, misdiagnosis can lead to negative appendectomy which, despite improvements in detailed medical anamnesis, physical examinations, laboratory examinations, and radiological imaging methods, has an incidence rate of 8.47% in the United States [3]. In addition, the perspective of physicians toward malpractice in treating appendicitis is important, as it is one of the more common diseases cited in malpractice cases. In one of three legal cases, especially in suits against emergency room (ER) physicians, the decision is against the physician and results in financial judgments [4]. Therefore, physicians are accountable for the accuracy of the diagnosis, the delay in recommending surgery, radiation risks to the patient from computed tomography (CT), the diagnostic success rates of clinical scoring systems, and laboratory parameters. Advancements in imaging modalities, such as ultrasound, tomography, and magnetic resonance imaging, are promising, but they are not completely adequate. Moreover, these methods can be expensive and are sometimes not readily available. Also, their use is limited in certain situations such as patients with contrast nephropathy and contrast allergy. Thus, there is still the need for a laboratory specimen for acute appendicitis that is inexpensive, readily available, quick, and able to provide high sensitivity and specificity rates. Mean platelet volume (MPV) and platelet distribution width (PDW) are two complete blood count assay parameters routinely used in the ER. MPV and PDW are biomarkers that show platelet activation and are associated with systemic inflammatory responses [5-6]. Few studies in the literature evaluate the association of MPV and PDW with acute appendicitis, and the results of these studies are contradictory [7-9]. In this study, we investigated the role of MPV and PDW as potential biomarkers in the diagnosis of acute appendicitis.

## Materials and methods

Our study was carried out retrospectively in the Department of Emergency Medicine at the Derince Training and Research Hospital in Kocaeli, Turkey. Within the scope of the study, the medical records of patients who underwent an appendectomy from January 2012 to January 2015 were retrospectively scanned. Our work was approved by the ethics committee of Kocaeli University (Approval number: KouKaek 2015/83). Patients older than age 15 years who reported abdominal pain and had an operation with a diagnosis of acute appendicitis were included in the study. We excluded patients referred from another center and those with chronic infections, comorbid diseases (e.g., cardiac, respiratory, renal, endocrine, vascular, cancer) and hematologic diseases. We also excluded patients who received blood transfusions in the previous year; used nonsteroidal anti-inflammatory medications, anticoagulant medications, and oral contraceptives; those with liver disease; and those with missing data in their files. Patients were divided into three groups according to their pathology results: non-appendicitis (i.e., healthy appendices and reactive lymph node hyperplasia), uncomplicated appendicitis (i.e., appendicitis without peritonitis and phlegmonous appendicitis), and complicated appendicitis (i.e., perforated appendicitis, appendicitis accompanied by peritonitis, plastron appendicitis, and/or necrotizing appendicitis). Peripheral venous blood was collected in ethylenediaminetetraacetic acid (EDTA)-containing tubes and analyzed in one hour. Complete blood count (CBC) and c-reactive protein (CRP) values were recorded. An Abbott Architect C16000 analyzer (Abbott Diagnostics, USA) was used to assess CRP measurements, and the Cell-Dyn 3700 Haematology analyzer (Abbott Diagnostics, USA) to assess CBC measurements. The reference intervals were 4.5 × 109/L to 10.3 × 109/L for the WBC, 1.4 to 6.2 × 109/L for the neutrophil count (NC), 7.4 to 10.4 fL for the MPV, 8% to 18 % for PDW, and 0.76 to 28.5 nmol/L for CRP.

Statistical analysis

The distribution of data was determined by the Kolmogorov-Smirnov test. Continuous variables were expressed as mean ± standard deviation and categorical variables as frequency and percent. Continuous variables were compared using Mann-Whitney U-test, and categorical variables were compared using Pearson’s Chi-square test for groups. Additionally, receiver operating characteristic (ROC) curve analysis was used to evaluate biomarker accuracy. A p-value of less than 0.05 was regarded as statistically significant. Statistical analysis was performed using SPSS Statistics for Windows, Version 17.0. (SPSS, Inc., Chicago).

## Results

A total of 322 patients with a diagnosis of appendicitis who received an appendectomy were included in the study. Seventy-five patients (23.3%) were ultimately excluded according to the exclusion criteria or as a result of data missing from their medical files. The mean age of the 247 included patients was 32 ± 13 years, and 133 of the patients were male (53.8% male, 46.2% female). When comparing Group 1 and others, we found that negative appendectomy was more common in women, and WBC and NC were statistically significant (Table 1).

**Table 1:** Comparison of sociodemographic characteristics and laboratory testings between appendicitis and non-appendicitis groups CRP, C-reactive protein; MPW, mean platelet volume; NC, neutrophil count; PDW, platelet distribution width; WBC, white blood cell

Variables	Non-Appendicitis (Group 1; n = 39)	Acute Appendicitis (Group 2+3; n = 208)	p
Gender	n (%)	n (%)	0.048
Male	14 (36%)	119 (57%)	
Female	25 (64%)	89 (43%)	
	Mean ± SD	Mean ± SD	
Age in years	32 ±14	32 ± 13	0.930
WBC (x10^9^/L)	11.98 ± 3.43	13.90± 4.05	0.060
MPV (fL)	7.89 ± 1.32	7.78± 1.12	0.594
PDW (%)	17.38 ± 1.17	17.16± 0.98	0.216
NC (x10^9^/L)	8.77 ± 3.3	10.82± 4.05	0.030
Neutrophil %	71.46 ± 10.43	76.12 ± 10.96	0.015
CRP (nmol/L)	38.46 ± 53.82	41.17 ± 53.66	0.773

Moreover, receiver operating characteristic (ROC) analysis revealed that only WBC and NC were above 0.600 (Table 2).

**Table 2:** AUC of differences between appendicitis and non-appendicitis groups AUC, area under receiver operating characteristic; CI, confidence interval; CRP, C-reactive protein; MPW, mean platelet volume; NC, neutrophil count; PDW, platelet distribution width; WBC, white blood cell

Variables	AUC	95% CI	p
WBC	0.645	0.558-0.732	0.004
NC	0.647	0.561-0.733	0.004
MPW	0.489	0.390-0.587	0.826
PDW	0.446	0.347-0.545	0.286
CRP	0.511	0.418-0.605	0.823

The recommended cutoff values for the parameters used in the discrimination of each group and the performance of these values are shown in Table 3.

**Table 3:** AUC of differences between appendicitis and non-appendicitis groups AUC, area under receiver operating characteristic; CRP, C-reactive protein; MPV, mean platelet volume; NEU, neutrophil count; PDW, platelet distribution width; WBC, white blood cell; PPV, positive predictive value; NPV, negative predictive value; pLLR, positive likelihood ratio; nLLR, negative likelihood ratio

Variables	Cut-off value	Sensitivity (%)	Specifity (%)	PPV	NPV	pLLR	nLLR	AUC	Accuracy(%)
WBC	15.35	45.6	92.3	0.96	0.21	5.92	0.58	0.645	44.5
NC	11.15	45.2	79.5	0.92	0.21	2.2	0.68	0.647	50.6
MPV	8.05	34.1	69.2	0.85	0.16	1.1	0.95	0.489	39.6
PDW	18.05	14.9	84.6	0.83	0.16	0.97	1.01	0.446	25.9
CRP	47.2	41.7	79.5	0.89	0.18	2.03	0.73	0.511	39.2

When comparing Group 2 and Group 3, no parameters were statistically significant (Table 4).

**Table 4:** Comparison of sociodemographic characteristics and laboratory testings between uncomplicated appendicitis and complicated appendicitis groups CRP, C-reactive protein; MPV, mean platelet volume; NC, neutrophil count; PDW, platelet distribution width; WBC, white blood cell

Variables	Uncomplicated appendicitis (Group 2; n = 169)	Complicated appendicitis (Group 3; n = 39)	p
Gender	n (%)	n (%)	
Male	96 (57%)	23 (59%)	0.371
Female	73 (43%)	16 (41%)	
	Mean ± SD	Mean ± SD	
Age in years	32 ± 14	34 ± 11	0.419
WBC (x10^9^/L)	13.83 ± 4.10	14.16 ± 3.91	0.648
MPV (fL)	7.80 ± 1.19	7.70 ± 0.80	0.541
PDW (%)	17.17 ± 1.04	17.12 ± 0.69	0.786
NC (x10^9^/L)	10.75 ± 4.07	11.13 ± 4.00	0.595
CRP (nmol/L)	39.38 ± 54.54	48.93 ± 49.54	0.318

The ROC analysis, revealed that no parameters had an area under the curve higher than 0.600 (Table 5).

**Table 5:** AUC of differences between uncomplicated appendicitis and complicated appendicitis groups AUC, area under receiver operating characteristic; CI, confidence interval; CRP, C-reactive protein; MPV, mean platelet volume; NC, neutrophil count; PDW, platelet distribution width; WBC: white blood cell

Variables	AUC	95% CI	p
WBC	0.524	0.424-0.623	0.645
NC	0.525	0.425-0.625	0.627
MPV	0.501	0.410-0.593	0.979
PDW	0.524	0.430-0.619	0.638
CRP	0.578	0.477-0.680	0.128

When the normal cutoff values of the laboratory parameters were evaluated in terms of acute appendicitis, the highest sensitivity was observed in the NC, while the highest specificity values were in the MPV (Table 6).

**Table 6:** Sensitivity and specificity numbers of the laboratory parameters CRP, C-reactive protein; MPV, mean platelet volume; NC, neutrophil count; PDW, platelet distribution width; WBC, white blood cell

Variables	Non-appendicitis (n=39) Negative test/Positive test	Appendicitis (n=208) Negative test/Positive test	Sensitivity	Specificity
WBC	10/29	39/169	81.2%	25.6%
CRP	10/29	46/162	77.8%	25.6%
NC	10/29	33/175	84.1%	25.6%
MPV	27/12	71/137	65.8%	69.2%
PDW	23/16	110/98	47.1%	58.9%
WBC and NC	8/31	28/180	86.5%	20.5%
WBC and CRP	3/36	9/199	95.7%	7.7%
WBC, CRP and NC	2/37	9/199	95.7%	5.1%

When the combinations of laboratory tests were examined, 95.7% sensitivity was reached in the combination of WBC and CRP; adding NC to this combination did not change sensitivity values.

Both MPV and PDW values were lower in patients with acute appendicitis than in non-appendicitis cases, and even lower in complicated appendicitis cases, but none of these differences were statistically significant. The performance of the MPV and PDW in the ROC curve was not a definite diagnostic indicator in clinical use. Using the WBC and CRP combination in the differentiation of patients with and without acute appendicitis produced high sensitivity values, and adding the NC to this combination did not lead to an increase in sensitivity.

## Discussion

Many studies suggest MPV may be related to diseases over a wide spectrum. Park et al. found that MPVs were significantly lower in metabolic syndrome patients [10]. However, there are studies that show no significant differences in these patients, while other studies show that they have significantly higher MPVs [11-12]. Although most studies show statistically significantly lower MPV in acute appendicitis patients, there are studies which show no significant difference, and one study shows statistically higher MPV [8-9,13]. In our study, MPVs were not significantly different in acute appendicitis patients. Moreover, there are various opinions about the factors affecting MPV levels in different diseases. Elevated interleukin-6 levels in diseases accompanied by inflammation activate megakaryocytes in the bone marrow, leading to the involvement of younger, relatively larger thrombocytes in circulation. This results in higher MPV. This is particularly common in periods when diseases such as ulcerative colitis and rheumatoid arthritis show low levels of activity. Gasparyan et al. stated that MPVs tend to decrease during periods of high activity in inflammatory diseases, and hence, Danese et al. suggest that, when the inflammatory activity is high, large, activated platelets get sequestrated and destroyed in the inflammatory zone, and small platelets become dominant [14-15].

The literature suggests that the very different outcomes between MPV levels in acute appendicitis may be valid in all views. Depending on which of these two mechanisms predominate, MPV levels may result in meaningful differences, such as low and high values, or no significant difference as found in our study. In such a case, it is expected that platelets of different sizes will be in circulation at the same time, causing a significant increase in PDW.

Given that we found no significant difference between PDW across the study groups, it is useful to look at other possible factors that may affect MPV such as obesity, hypertension, smoking, hyperlipidemia, atrial fibrillation, rheumatologic diseases, inflammatory diseases, metabolic syndrome, and diabetes. Study participants may be unaware of these factors [16]. Also, the method of measuring MPV is important because the platelets swell in the presence of EDTA but shrink in citrated tubes [17]. However, anticoagulant use is necessary for accurate measurement. The waiting time prior to the measurement is another important factor. Dastjerdi et al. reported that, for the EDTA tube, the measurement should be made within 60 minutes, while Lance et al. reported the wait time prior to measurement should be 120 minutes for EDTA and 60 minutes for citrated tubes [18-19]. Currently, many laboratories, including ours, use EDTA for anticoagulation, as recommended by the International Council for Standardization in Haematology. In the literature, some studies on MPV association with acute appendicitis reported the use of EDTA for anticoagulation [8,20]. Other studies provided no anticoagulant information in their methodologies [21-22]. In addition, the sample wait time prior to analysis was less than two hours in one study and less than one hour in two studies [7-8,20]. Other studies provide no information on similar analysis times [9,21]. Panova-Noeva et al. found that age, cardiovascular risk factors, hypertension, and hyperglycemia were associated with higher MPV in men, and the same were associated with oral contraceptive use and menstruation in women [22]. They showed that seven single nucleotide polymorphisms (rs342293, rs7961894, rs12485738, rs649729, rs342251, rs17568628, and rs4774471) in women are associated with higher MPV and that four single nucleotide polymorphisms (rs342293, rs7961894, rs10876550, and rs342251) in men are also associated with higher MPVs.

The variety of factors that can affect MPV levels has led to a wide spectrum of results in the literature concerning the relationship between acute appendicitis and MPV involvement. This variability also makes platelet indices poor markers in diagnosing acute appendicitis. Many studies have evaluated the importance of CRP, leukocyte, and neutrophil count and percentage for the diagnosis of acute appendicitis; these studies show that none of these completely exclude the diagnosis of acute appendicitis. When we look at specific rates, there are published studies in which the results are 65% to 85% for WBC sensitivity and 32% to 82% for specificity [23]. Also, in studies in which CRP was evaluated, the sensitivity rates are reported as 65% to 85% with specificity rates of 59% to 73% [24-25]. For NC, some studies show 71% to 89% for sensitivity and 48% to 80% for specificity [24,26-27]. In our study, the rates we found for WBC, CRP, and NC were slightly lower than those found in the literature regarding specificity values, but they generally agree with the literature, and, as in other studies, we cannot rule out acute appendicitis from the rates in our study. One point to note here is that complications of acute appendicitis, such as peritonitis or abscess formation, are more serious than complications of a negative appendectomy [28]. Therefore, tests that reveal high sensitivity rates are very important. One way to achieve high sensitivity rates is to combine tests but to do this, it is necessary to sacrifice the specificity values. When we combined WBC and CRP values in our study, we attained very high sensitivity rates, but the specificity values, already low as a result of the combination, dropped even lower. Furthermore, when we added neutrophil values, there was no increase in sensitivity rates. There are studies which show that healthy WBC and CRP levels in a patient exclude the possibility of acute appendicitis [29,30]. However, in our study, both WBC and CRP levels were within reference ranges in nine of 208 appendicitis patients. Thus, although appendicitis appears to be unlikely in patients with healthy WBC and CRP levels if they have other clinical symptoms and findings that indicate appendicitis, longer follow-up times and, if there is not enough improvement in clinical reevaluations, advanced imaging methods such as CT are appropriate for these patients.

The primary limitations of our study are its retrospective nature and that it was conducted at only one medical center. However, our samples were all analyzed in the same analyzer, eliminating inter-analyzer variables. In addition, we excluded patients with abdominal pain suspected of being caused by appendicitis yet did not undergo a surgical operation. However, the lack of an endpoint in patient classification in studies related to the use of biomarkers in the diagnosis of appendicitis is one of the more important factors influencing the consistency of the results of these studies. In our study, the participation of only those patients receiving surgery removed this problem. 

## Conclusions

MPV and PDW values in the differential diagnosis of acute appendicitis were far from being significant predictors of clinical decisions. Acute appendicitis in patients with healthy WBC and CRP values is possible, albeit rare. These patients may benefit from longer follow-up periods, and, if such patients have not improved after a period of clinical observation, further diagnostic imaging methods are strongly recommended.
